# Deep Common Semantic Space Embedding for Sketch-Based 3D Model Retrieval

**DOI:** 10.3390/e21040369

**Published:** 2019-04-04

**Authors:** Jing Bai, Mengjie Wang, Dexin Kong

**Affiliations:** 1 School of Computer Science and Engineering, North Minzu University, Yinchuan 750021, China; 2 Ningxia Province Key Laboratory of Intelligent Information and Data Processing, Yinchuan 750021, China

**Keywords:** sketch-based retrieval, 3D model retrieval, deep common semantic space embedding, metric learning, cross-entropy

## Abstract

Sketch-based 3D model retrieval has become an important research topic in many applications, such as computer graphics and computer-aided design. Although sketches and 3D models have huge interdomain visual perception discrepancies, and sketches of the same object have remarkable intradomain visual perception diversity, the 3D models and sketches of the same class share common semantic content. Motivated by these findings, we propose a novel approach for sketch-based 3D model retrieval by constructing a deep common semantic space embedding using triplet network. First, a common data space is constructed by representing every 3D model as a group of views. Second, a common modality space is generated by translating views to sketches according to cross entropy evaluation. Third, a common semantic space embedding for two domains is learned based on a triplet network. Finally, based on the learned features of sketches and 3D models, four kinds of distance metrics between sketches and 3D models are designed, and sketch-based 3D model retrieval results are achieved. The experimental results using the Shape Retrieval Contest (SHREC) 2013 and SHREC 2014 datasets reveal the superiority of our proposed method over state-of-the-art methods.

## 1. Introduction

With the rapid development of computer hardware, 3D data acquisition, and shape modeling technologies, 3D models have become increasingly useful in various fields, and as a result, 3D model retrieval and reuse has received increasing attention. There are two key challenges to retrieving a 3D model: creating a model that is simple enough for novice users and producing a matching algorithm that is robust enough to work for arbitrary polygonal models [1]. Accordingly, example-based 3D model retrieval has attracted widespread attention. Unfortunately, although example-based 3D model retrieval is intuitively straightforward, it is difficult to achieve [2]. Photo input is another natural method for 3D model retrieval when user has the target 3D object. However, user cannot obtain the 3D object in many applications, such as the conceptual design stage of a new product. Instead, due to its intuitive nature and convenience, sketch-based 3D model retrieval plays a practical role in many applications, including sketch-based rapid modeling, recognition, 3D printing and 3D animation production.

**Figure 1:**
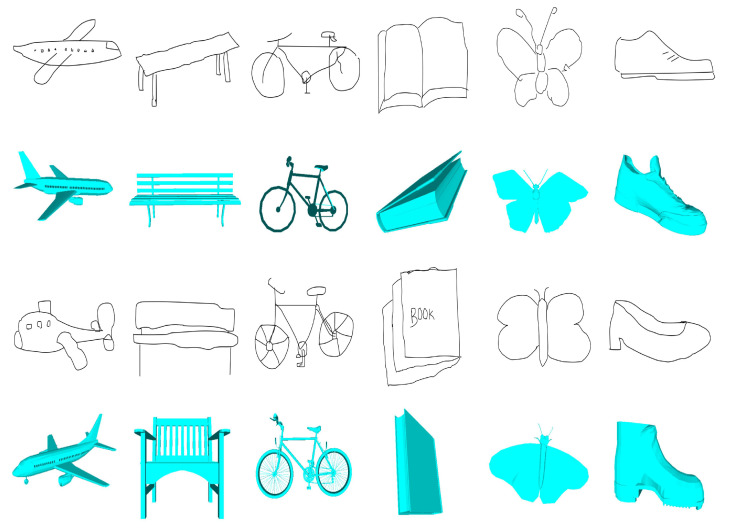
Sketches and the corresponding 3D models.

However, sketch-based 3D model retrieval is more challenging than example-based retrieval. This difficulty is due to two main characteristics of sketch-based 3D model retrieval: (1) large interdomain visual perception discrepancies between sketches and 3D models (as Figure 1 shows, 3D models are precise and informative, while sketches are concise and abstract) and (2) large intradomain visual perception diversities for sketches of the same object, as shown in Figure 1. This diversity occurs because a sketch is a subjective expression of an object that is influenced by factors such as the thought processes, domain background and hand-drawing habits of the person creating the sketch. The above two factors are related to each other and lead to difficulty in sketch-based 3D model retrieval.

Although there are large interdomain visual perception discrepancies and significant intradomain visual perception diversity for sketch-based 3D model retrieval, the 3D models and sketches of the same class share common semantic content. Motivated by these findings, we propose a novel approach for sketch-based 3D model retrieval by constructing a deep common semantic space embedding using a triplet network (DCSSE). First, 3D models are described by a group of views. Then, a common modality space is constructed by translating one mode to another based on cross entropy. Finally, a common semantic space embedding is learned based on a triplet network, and the essential features of sketches and 3D models are generated simultaneously by synthetically considering the two domains. The retrieval experiments on Shape Retrieval Contest (SHREC) 2013 and SHREC 2014 demonstrate the effectiveness of our approach.

This paper makes the following contributions. (1) A cross-entropy-based common modality space is constructed for sketches and 3D models, which reduces interdomain visual perception discrepancies. (2) A DCSSE is generated between sketches and 3D models via synthetical consideration of the sketches, the 3D models and their shared semantics. (3) A novel combination of deep metric learning with cross-domain transformation is adopted, which has more relaxed constraints and is more consistent with the two characteristics of sketch-based 3D model retrieval. (4) The approach outperforms all state-of-the-art methods on two large benchmark datasets.

## 2. Related Work

At present, sketch-based 3D model retrieval is the most concerned hot spot in the field of 3D model retrieval. Researchers have proposed a variety of methods for sketch-based 3D model retrieval [3,4,5,6]. Earlier methods such as Histogram of Gradient [7], Gabor local line [8], View Context [9,10], part-based features [11,12], cross-domain manifold ranking (CDMR) [13], Shape2Vec [14] and composite features [15] algorithms have extracted handcrafted features for sketches and 3D models. However, due to the complexity of 3D models and the large interdomain visual perception discrepancies, these methods cannot objectively and reasonably capture the essential features of 3D models and sketches, and consequently, their accuracies are insufficient.

With the wide application of deep learning in computer vision, several deep feature learning-based methods are proposed. As 3D model representation is unstructured, it cannot be directly inputted into the deep learning model. Some of these methods describe 3D models using low-level feature vectors, such as physics-constrained deep neural network (PCDNN) [16], deep correlated metric learning (DCML) [17], deep correlated holistic metric learning (DCHML) [18], and semantic embedding space (SEM) [19], and then input the vectors into the deep learning model to generate the final features. These methods try to automatically learn and construct the features of complex models; however, they lose original information when extracting low-level features and fail to make full use of the characteristics of the deep learning algorithm. In addition, these methods do not consider the relationship between the sketches and the 3D models when extracting their features, resulting in unsatisfactory retrieval accuracies.

Another kind of deep feature learning-based method for sketch-based 3D model retrieval describes 3D models using a group of projected views, and then separately adopts two convolutional neural networks (CNNs) for the views and sketches, finally combines them by constructing the specific loss between the features of the two domains using methods such as the Siamese network (Siamese) [20], the learned Wasserstein barycentric representation (LWBR) [21], deep cross-modality adaptation (DCA) [22], and multiview attention network (MVAN) [23]. These methods have achieved state-of-the-art performance. However, the sketches are generally quite abstract with large local and global deviations from the original model [3]. As a result, any methods that extract features of views and sketches separately fail to explore the related features between the sketches and the 3D models. In addition, these methods construct a metric network by minimizing the loss to compel the features of the sketches and the 3D models of the same class to be identical or almost identical, which may be too restrictive for heterogeneous data.

## 3. Proposed Method

**Figure 2:**
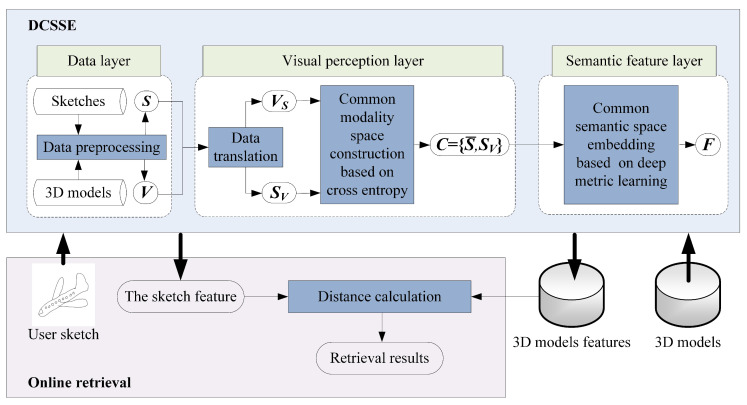
Framework of our proposed approach.

As illustrated in Figure 2, the proposed approach consists of two parts: the DCSSE model and online retrieval.

(1) DCSSE is composed of three layers: the data layer, the visual perception layer and the semantic feature layer. The inputs of DCSSE are the labeled 3D model dataset $M_{i}=\left\{M_{i},1\leq i\leq n_{m}\right\}$ and the sketch dataset $S=\left\{S_{i},1\leq i\leq n_{s}\right\}$ , where $n_{m}$ is the number of 3D models and $n_{s}$ is the number of sketches. In the data layer, a 3D model $M_{i}$ can be represented as *l* views $\left\{V_{i}^{j},1\leq j\leq l\right\}$ by data preprocessing, which ensures both the inputted 3D models and the sketches occupy 2D space and share the same data form. Then, in the visual perception layer, data translation is used to construct a common modality space based on cross entropy, impelling all inputted data to share a similar visual perception. Furthermore, in the semantic feature layer, for any 3D model $M_{i}$ and any sketch $S_{i}$, their feature embeddings $\left\{f\left(V_{i}^{j}\right),1\leq j\leq l\right\}$ and $f\left(S_{i}\right)$ can be generated after deep metric learning, and a common semantic space ***F*** is constructed.
(2) The online retrieval: Based on DCSSE, we can further extract the features of the user sketch and 3D models, calculate the distance between the user sketch and 3D models and return the retrieval results.

In the remainder of this section, we will elaborate on each step.

### 3.1. The Data Layer: Intradomain Data Preprocessing

Given the 3D model dataset *M* and the sketch dataset S, the purpose of data preprocessing in the data layer is to uniformly convert the data of the two different modalities into 2D images with the same data form.

#### 3.1.1. 3D Model Preprocessing

For the 3D model dataset *M*, a mapping function $\phi_{m}:M\to R^{m\times m\times l}$ is established and a multiview representation set $V=\left\{\phi_{m}\left(M_{i}\right)=\left\{V_{i}^{j},1\leq j\leq l,V_{i}^{j}\in R^{m\times m}\right\},1\leq i\leq n_{m}\right\}$ is obtained by 3D model preprocessing, where *l* is the number of views (images) and m×m is the size of the views. Comparing all kinds of multiview 3D model retrieval algorithms, we can see that the view-rendering method proposed by Multi-view Convolutional Neural Networks (Su-MVCNN) is excellent [24,25]. Therefore, in this paper, we use this method to construct a multiview representation of a given 3D model.

**Figure 3:**
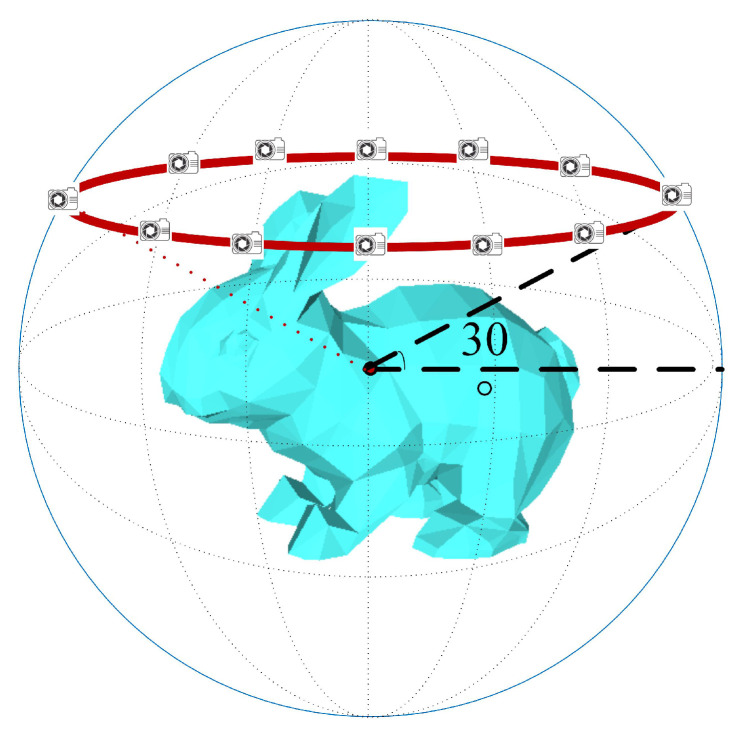
Multiview rendering of 3D models.

Taking 12 views as an example, the multiview rendering of a 3D model is shown in Figure 3. First, the 3D model is normalized into a unit sphere via translation and scaling. Second, as shown in Figure 3, along the red circle on the unit sphere surface, 12 virtual cameras are set at an interval of 30 degrees, and the cameras are positioned to point toward the sphere center. Finally, each view is rendered using the Phong reflection model [26].

As shown in Figure 3, because the views are uniformly located in different views of the 3D model, there is a strong mutual relationship between them. In addition, there is strong complementarity and low correlation between views; thus, the multiview representation obtained by this method constitutes a relatively complete description of the 3D model.

#### 3.1.2. 2D Sketch Preprocessing

For the sketch dataset S, a mapping function $\phi_{s}:S\to R^{m\times m}$ is established and a 2D image $\bar{S}=\{\phi_{s}\left(S_{i}\right)=\bar{S_{i}},\bar{S_{i}}\in R^{m\times m},1\leq i\leq n_{s}\}$ is obtained by sketch preprocessing, where m×m is the size of the output images. Here, we directly use the bilinear interpolation algorithm to complete the size transformation of the sketch image.

### 3.2. The Visual Perception Layer: Interdomain Data Translation

Although both the 3D models and the hand-drawn sketches have been represented in a 2D space with the same image sizes by intradomain data preprocessing, they are significantly different in terms of visual perception. Specifically, the multiview representations are accurate and informative, while the hand-drawn sketches are concise and abstract. To further narrow the interdomain differences and highlight the commonality of categories, we analyze the characteristics of views and sketches, achieve sketch-to-view and view-to-sketch interdomain translation, evaluate translation validity using cross entropy, and construct a 2D common modality space in this subsection.

#### 3.2.1. View-To-Sketch Translation

Since views contain complete visual information from a certain perspective of a 3D model and hand-drawn sketches mainly contain outline information of the 3D model, the translation from view-to-sketch can be accomplished by choosing a reasonable edge extraction algorithm. In this work, the model in [8], which detects Canny edges on the depth buffer (also known as the z-buffer), is used to translate the multiview representation set V to the extended sketch set $S_{V}=\{\tau_{v-s}\left(V_{i}^{j}\right),1\leq i\leq n_{m},1\leq j\leq l\}$.

#### 3.2.2. Sketch-To-View Translation

Compared with the views of the 3D model, the information contained in sketches is abstract, finite, and ambiguous, making it extremely difficult to generate high-quality views directly from sketches. In recent years, GAN [27], the generative adversarial network, has been proposed for high-quality image-to-image translation. Accordingly, inputting standardized sketch set $\bar{S}$ without any paired data, we achieve sketch-to-view translation and generate the extended view set $V_{S}=\{\tau_{s-v}\left(\bar{S_{i}}\right),1\leq i\leq n_{s}\}$ using CycleGAN [28].

#### 3.2.3. Construction of 2D Common Modality Space Based on Cross Entropy

**Figure 4:**
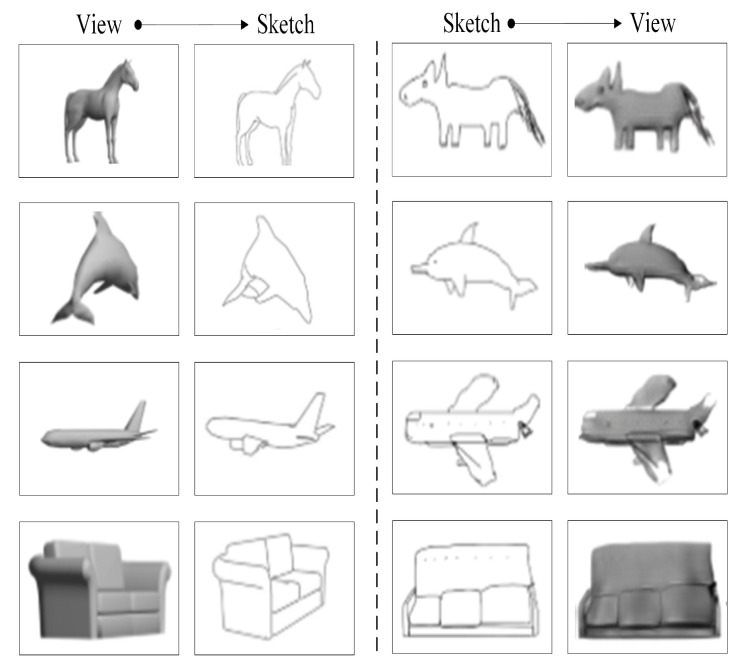
Translation results of the view-to-sketch and sketch-to-view translations.

Some translation results of the view-to-sketch and sketch-to-view translations using the methods mentioned in Section 3.2.1 and Section 3.2.2 are shown in Figure 4. Compared to the original sketches, the sketch translated from a given view loses some of the local detail information, such as the eyes of the dolphin; compared to original views, the view translated from a given sketch has problems with missing or additional texture information. In conclusion, neither translation is perfect.

To evaluate which kind of translation is more effective for the construction of the 2D common modality space to support cross-domain retrieval, cross-entropy [29], a metric of the difference between two probability distributions, is introduced to evaluate the two kinds of translation. Specifically, the gray-level histograms of images are used as statistical features. Let *r* = 0, …, 255 be the gray value, V(k,r) be the average probability value of all views corresponding to the k-class 3D models in the database when their gray value is *r*, $S_{V}(k,r)$ be the average probability value of all sketches translated from views of the k-class 3D models when the gray value is *r*, S(k,r) be the average probability value of all k-class sketches in the database when their gray value is *r*, and $V_{S}(k,r)$ be the average probability value of all views translated from *k*-class sketches when the grayscale value is *r*. The cross entropy of view-to-sketch translation is defined as follows:

(1) $$H(S,S_{V})=\sum_{k=1}^{c}\sum_{x=0}^{255}S\left(k,r\right)\log\frac{1}{S_{V}\left(k,r\right)}$$

where *c* is the number of sketch classes in the database (equal to the number of 3D model classes). The cross entropy of sketch-to-view translation is defined as follows:

(2) $$H(V,V_{S})=\sum_{k=1}^{c}\sum_{x=0}^{255}V\left(k,r\right)\log\frac{1}{V_{S}\left(k,r\right)}$$

where *c* is the number of sketch classes in the database (equal to the number of 3D model classes).

**Figure 5:**
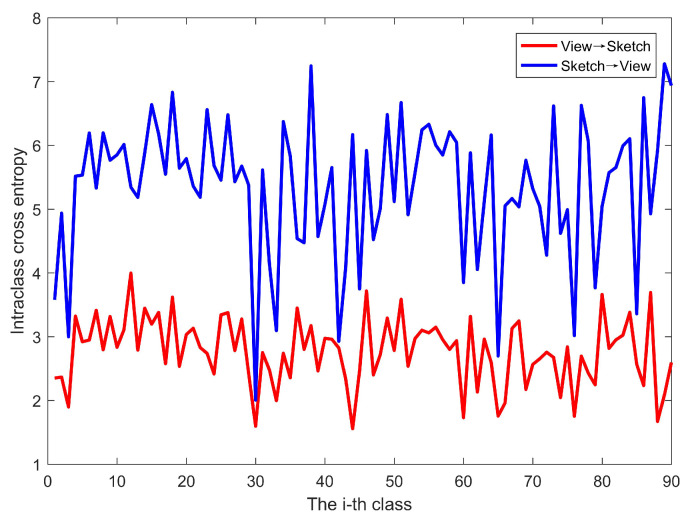
Comparison of intra-class cross entropy.

Figure 5 shows the intraclass cross entropy values of the view-to-sketch translation and sketch-to-view translations on the SHREC 2013 dataset. It can be seen that, regardless of the class to which the images belong, the cross-entropy values of the view-to-sketch translation are smaller than those of the sketch-to-view translation, demonstrating that the sketches translated by views can better simulate the probability distribution of the original sketches. We performed the same experiment on the SHREC14 dataset, and the results are the same. Based on this discovery, we choose the view-to-sketch translation to construct the common modality space $C\;=\;\bar{S}\cup S_{V}$ for sketches and 3D models to impel the inputted data to share a similar visual perception.

### 3.3. The Semantic Feature Layer: Cross-Domain Common Semantic Space Embedding

**Figure 6:**
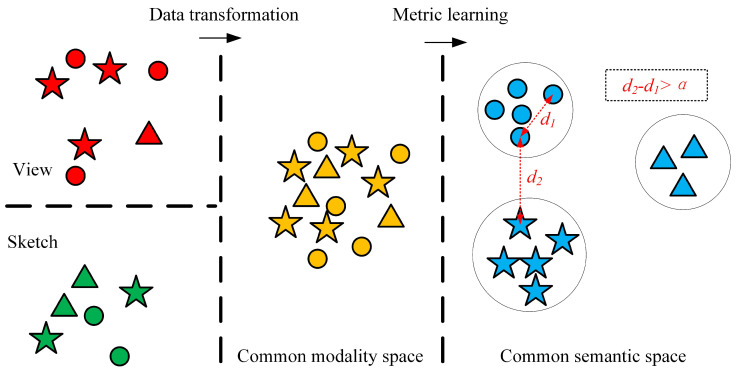
Illustration of the common modality space and the common semantic space. The color represents the modality and the shape represents the class. Input data come from two modalities (i.e., sketches and views of 3D shapes). First, the two types of input data are translated into a common modality space, where data of different modalities share a common space; however, data in different classes are mixed and indistinguishable. Then, a common semantic space is constructed using deep metric learning, where the same kinds of samples are nearer and different kinds of samples are farther.

As shown in Figure 6, the modality sharing between 3D models and sketches has been constructed through the interdomain data translation; however, data of different classes in this common modality space are mixed and indistinguishable. To solve this issue, a deep metric learning model is introduced to build a common feature space, narrowing the distance of the same class and widening the sample distance of different classes.

**Figure 7:**
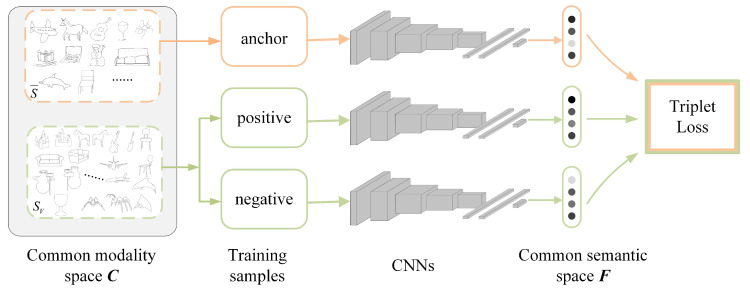
Construction of the common semantic space ***F*** based on triplet convolutional neural networks (CNNs).

Typical deep metric learning includes Siamese and triplet networks. Compared with the Siamese network with contrastive loss, the triplet network [30] learns a ranking function for retrieval, which has more relaxed constraints. Therefore, considering the large interdomain visual perception discrepancies and the significant intradomain visual perception diversity for sketch-based 3D model retrieval, the triplet network with deep ranking loss is chosen to construct a common semantic space ***F*** for cross-modality retrieval. As shown in Figure 7, the details are as follows:

(1) Selection of triples. The anchor training samples in the triples are selected from the normalized sketch dataset $\bar{S}$ , and positive and negative training samples are selected from the extended sketch dataset $S_{V}$. Positive samples must have the same class as a given anchor training sample, while negative training samples must have a different class.
(2) Construction of the CNNs. Sketch-based 3D model retrieval is very complex, but the information in each sketch is relatively sparse. Therefore, taking AlexNet as the prototype [31], a medium-sized CNN is constructed. This network consists of eight layers; the first five layers are convolutional layers, the middle two layers are fully connected layers, and the last layer is the feature output layer Feat. The details are shown in Table 1.
(3) Establishment of the loss function. Given the $i^{th}$ triplets $x_{i}^{a}$, $x_{i}^{p}$, $x_{i}^{n}$ of the objects, we take $x_{i}^{a}$ to be the anchor sample, $x_{i}^{p}$ to be the same class as $x_{i}^{a}$, $x_{i}^{n}$ to be of a different class, $f\left(x\right)$ to be the embedded feature representation of the network, and ***F*** to be the embedded feature space. The metric function should satisfy the following:
  (3) $$\begin{array}{c} {∥f\left(x_{i}^{a}\right)-f\left(x_{i}^{p}\right)∥}_{2}^{2}+\alpha <{∥f\left(x_{i}^{a}\right)-f\left(x_{i}^{n}\right)∥}_{2}^{2}\\ \forall f\left(x_{i}^{a}\right),f\left(x_{i}^{p}\right),f\left(x_{i}^{n}\right)\in F \end{array}$$
  where α is the interval threshold and requires the minimum distance difference between the same class and different classes to be α.
  The corresponding loss function can be expressed as follows:
  (4) $$L\left(f\right)=arg\min\sum_{i}{\left({∥f\left(x_{i}^{a}\right)-f\left(x_{i}^{p}\right)∥}_{2}^{2}-{∥f\left(x_{i}^{a}\right)-f\left(x_{i}^{n}\right)∥}_{2}^{2}+\alpha \right)}_{+}$$
  Here, ${\left(g\right)}_{+}=0$ if g<0, otherwise ${\left(g\right)}_{+}=g$.
(4) Implementation.

**Data augmentation.** For each sketch and each view of the 3D models, the image is first resized to [256, 256]. Then, a 225×225 image is randomly cropped from the image, or its deformation is rotated 30 degrees forward and backward, with the per-pixel mean subtracted.

**Data shuffle.** Notably, to enhance the generalizability of the network, we randomly disrupted the order of the training data when generating training sets to prevent the same kind of training samples from appearing in a single batch during training.

**Training.** DCSSE is trained by the stochastic gradient descent (SGD) + Newton momentum method with a mini-batch size of 125. Here, SGD training is fast and can converge at a faster speed for large datasets; however, there is an instability problem. The introduction of Newton momentum can restrain oscillation and enhance the stability of network learning when the gradient direction before is different from that after iteration. The update formulas are as follows:

(5) $$\theta_{new}=\theta -\lambda \nabla \theta +\varepsilon v_{t-1}$$

where, $\theta_{new}$ is the set of updated parameters, θ is the set of original parameters, λ is the current learning rate, ∇θ is the gradient of the parameters at the current position, $v_{t-1}$ is the momentum accumulated in all previous steps, and ε is the weight of momentum. Here, the learning rate λ is initialized as 0.0001 and ε is set to 0.9. Furthermore, as in Formula (6), an algorithm of adaptive learning rates adjusts the network’s weight to enhance the convergence speed.

(6) λnew=λλ(1+γ∗α)k(1+γ∗α)k

Here, $\lambda_{new}$ is the learning rate, λ is the original learning rate, α is the number of iterations, and *k* and γ are parameters used to update the learning rate and are set to 0.75 and 0.0001, respectively.

### 3.4. Cross-Domain Distance Metric

Given a sketch $S_{i}\in S$ , let its feature be denoted as the d-dimensional vector $x=\left\{f\left(S_{i}\right)=\left(x_{1},x_{2},\ldots ,x_{d}\right),1\leq i\leq n_{s}\right\}$; given a 3D model $M_{k}\in M$, let its feature be the l×d dimensional matrix $y=\{y^{j}=f{(V}_{k}^{j})=\left(y_{1}^{j},y_{2}^{j},\ldots ,y_{d}^{j}\right),1\leq j\leq l,1\leq k\leq n_{m}\}$, where *l* is the number of views, $n_{s}$ and $n_{m}$ are the number of sketches and 3D models, respectively. The distance from the sketch $S_{i}$ to the 3D model $M_{k}$ can be denoted as D(x→y). Four kinds of distance metrics are proposed in this paper, as follows.

(1) D(x→y) is defined as the average distance from a sketch feature to all view features of a 3D model, calculated as follows:
  (7) $$D\left(x\to y\right)=\frac{\sum_{j}d(x,y^{j})}{l},1\leq j\leq l$$
(2) D(x→y) is defined as the minimum distance from a sketch feature to all view features of a 3D model, calculated as follows:
  (8) $$D\left(x\to y\right)=\min_{j}d\left(x,y^{j}\right),1\leq j\leq l$$

In Formulas (7) and (8), $d(x,y^{j})$ is the distance between the sketch feature vector *x* and the feature vector $y^{j}$ of the *j*th view of the 3D model. Here, both the Euclidean distance and the Wasserstein distance (earthmover’s distance) can be selected; they are calculated as follows:

Euclidean distance:

(9) $$d(x,y^{j})=\sqrt{\sum_{i=1}^{d}{\left(x_{i}-y_{i}^{j}\right)}^{2}}$$

Wasserstein distance:

(10) $$d(x,y^{j})=\sum_{i=1}^{d}\sum_{m=1}^{d}d_{im}f_{im}$$

where, $d_{im}$ is the spatial distance from $x_{i}$ to $y_{m}^{j}$ , and $f_{im}$ is the amount of soil moved from $x_{i}$ to $y_{m}^{j}$.

Four kinds of distance metrics, EUD_Ave, EUD_Min, EMD_Ave, and EMD_Min are acquired by substituting the Euclidean distance and the Wasserstein distance into the expressions (7) and (8) respectively. Accordingly, the similarity between a query sketch and all 3D models can be calculated and compared so that the sketch-based 3D model retrieval can be realized.

## 4. Experiments

### 4.1. Datasets and Evaluation Metrics

We conduct experiments on two large-scale sketch-based 3D model retrieval datasets, namely SHREC 2013 and SHREC 2014.

**SHREC 2013** [3,5]: This dataset is composed of 7200 sketches and 1258 3D models, divided into 90 classes. The number of 3D models in each class varies from 4 to more than 184, while the number of sketches for each class is equal to 80. In the experiment, 50 sketches per class are randomly selected for training and the remaining 30 sketches per class are used for testing; a total of 1258 3D models are targeted for retrieval.

**SHREC 2014** [4,6]: Compared with the SHREC 2013 dataset, this dataset has more classes and models. Specifically, this dataset has 13,680 sketches and 8978 3D models, divided into 171 classes. The number of 3D models in the different classes varies greatly from less than 10 to more than 300. The number of sketches for each class is also equal to 80, and for each class 50 sketches are for training, and 30 sketches are for testing.

To comprehensively evaluate the performance of the proposed method, we employ seven commonly adopted performance metrics in the 3D model retrieval field [5,6]: The precision-recall diagram, the nearest neighbor (NN), the first tier (FT),the second tier (ST), the E measures (E), the discounted cumulative gain (DCG) and the average precision (AP).

### 4.2. Comparison of Different Distances

In this section, we will compare the effects of the proposed retrieval method using four different distances. Table 2 shows the comparison results on the SHREC 2013 dataset. In Table 2, for all evaluation criteria, the method using the EUD_Ave distance outperforms the other methods. Through analysis, we found two reasons for this result: First, the average distance could fully consider the comprehensive information of all views’ features; and second, there is no direct comparison between the different feature components. Therefore, the Wasserstein distance is not as good as the Euclidean distance.

### 4.3. Comparison with the State-Of-The Art Methods

**Retrieval from the SHREC 2013 dataset.** In this subsection, the proposed method is tested on the SHREC 2013 dataset and compared with the state-of-the-art methods, including the cross domain manifold ranking method (CDMR) [13], the sketch-based retrieval method with view clustering (SBR-VC) [5], the spatial proximity method (SP) [32], Fourier descriptors on 3D model silhouettes (FDC) [5], the Siamese network (Siamese) [20], the deep correlated metric learning (DCML) [17], the learned Wasserstein barycentric representation method (LWBR) [21], and semantic embedding space (SEM) [19] methods.

**Figure 8:**
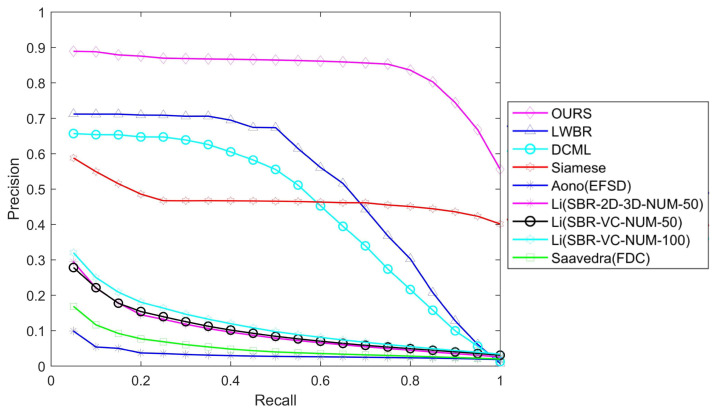
Precision-recall comparisons on the SHREC 2013 dataset

We use standard precision-recall curves to visualize our results. Figure 8 shows that our proposed method significantly outperforms the state-of-the-art methods. (1) Our method has the highest precision. For every recall value, our method gives better precision than the competing methods. On average, the precision values are 63%, 89%, and 77% higher than those of LWBR, DCML and Siamese, respectively. (2) The precision of our method is very stable: The precision-recall curve is closer to the horizontal line when the recall is less than 80%, and higher than 55% until the recall is 100%. This finding indicates that 80% of the related models within the target object’s class are returned as the top hits.

Except for the precision–recall curve, other standard metrics, including NN, FT, ST, E, DCG and AP, are also calculated and compared with the state-of-art methods. Table 3 shows the results on the SHREC 2013 dataset, which indicate that the proposed method is comparable to that of SEM [19] and has an evident advantage over other classical methods for every criterion.

**Figure 9:**
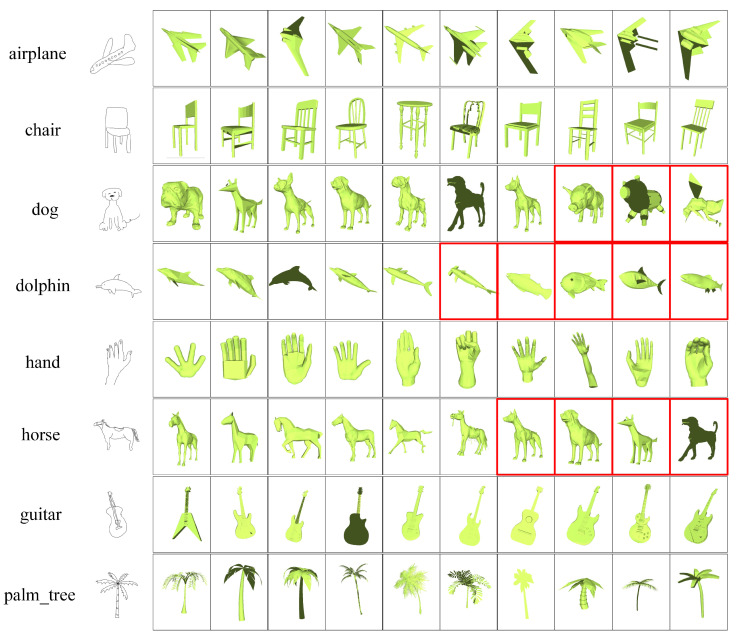
Some retrieval examples on the SHREC 2013 dataset.

Figure 9 presents some examples of sketch-based 3D model retrieval results using the proposed method. The query’s labels and sketches are listed on the left, and the top 10 retrieved models are listed on the right side in ascending order of distance. The correct results are framed in black, and the incorrect results are framed in red. For the classes of airplane, chair, hand, guitar and palm tree that all 10 retrieved models are correct, while for the classes of dog, dolphin and horse, the first few results retrieved are correct, and the last ones are incorrect. This finding is because the classes of dog, dolphin and horse contain only 7, 5 and 6 models, respectively.

**Retrieval from the SHREC14 dataset.** In this subsection, the proposed method is tested on the SHREC 2014 dataset and compared with the state-of-the-art methods, including BF-fGALIF [8], CDMR [13], SBR-VC [5], Siamese network [20], DCML [17], LWBR [21], SEM [19], BOF-JESC [33], and MVPR [34].

**Figure 10:**
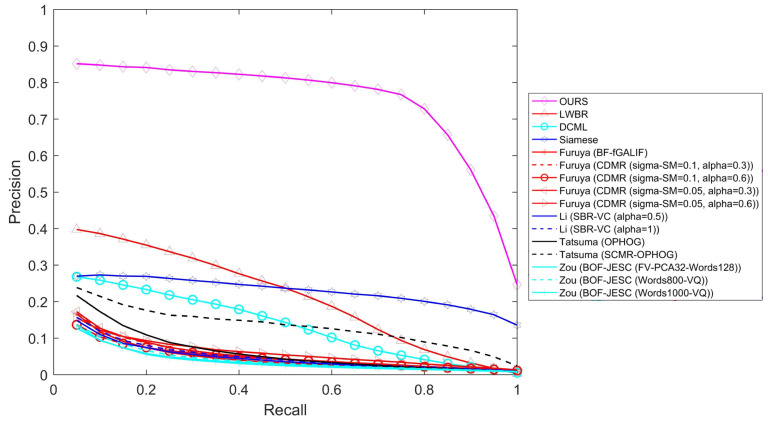
Precision–recall comparisons on the SHREC 2014 dataset.

Figure 10 shows the precision–recall curves. Obviously, the proposed method is still significantly better than other methods and very stable for every recall value. Table 4 shows performance comparisons of different evaluation criteria on the SHREC 2014 dataset and reveals that the proposed method also dominates for every criterion when using a large dataset.

## 5. Conclusions

In this paper, we presented an effective approach for sketch-based 3D model retrieval using deep common semantic space embedding (DCSSE). To reduce the visual perception gap between sketches and 3D models, we transformed the data of two different domains into one common modality based on cross entropy evaluation. Furthermore, we learned the features of the sketches and 3D models simultaneously via deep common semantic embedding. We trained the deep embedding using a triplet network according to the characteristics of the cross-domain data. Finally, four distance metrics are used to calculate the sketch query and 3D models in the database. The experiments on the large-scale datasets SHREC 2013 and SHREC 2014 demonstrate that the proposed approach is superior to state-of-the-art algorithms. 

## Figures and Tables

**Table 1:** Network structure of the constructed CNN. Abbreviations: Convolution (Conv), Rectified Linear Unit (Relu), Normalization (Norm), Pooling (Pool), Full connected layer (Fc), and Feature (Feat).

Layer	Filter Size	Stride	Pad	Feature Maps	Input Size	Output Size
Conv1	11×11	4	0	96	225×225×1	54×54×96
Relu1	–	–	–	–	54×54×96	54×54×96
Norm1	–	–	–	–	54×54×96	54×54×96
Pool1	3×3	2	0	96	54×54×96	27×27×96
Conv2	5×5	–	2	256	27×27×96	27×27×256
Relu2	–	–	–	–	27×27×256	27×27×256
Norm2	–	–	–	–	27×27×256	27×27×256
Pool2	3×3	2	0	256	27×27×256	13×13×256
Conv3	3×3	0	1	384	13×13×256	13×13×384
Relu3	–	–	–	–	13×13×384	13×13×384
Conv4	3×3	0	1	384	13×13×384	13×13×384
Relu4	–	–	–	–	13×13×384	13×13×384
Conv5	3×3	0	1	256	13×13×384	13×13×256
Relu5	–	–	–	–	13×13×256	13×13×256
Pool5	3×3	2	0	256	13×13×256	6×6×256
Fc6	–	–	–	–	6×6×256	4096
Relu6	–	–	–	–	4096	4096
Dropout6	–	–	–	–	4096	4096
Fc7	–	–	–	–	4096	4096
Relu7	–	–	–	–	4096	4096
Dropout 7	–	–	–	–	4096	4096
Feat	–	–	–	–	4096	200

**Table 2:** Retrieval performance comparisons using different distances on the SHREC 2013 dataset. The nearest neighbor (NN), the first tier (FT),the second tier (ST), the E measures (E), the discounted cumulative gain (DCG) and the average precision (AP). The best performance indicators are marked as bold.

–	NN	FT	ST	E	DCG	AP
EUD_Ave	**0.849**	**0.772**	**0.858**	**0.410**	**0.888**	**0.817**
EUD_Min	0.816	0.744	0.847	0.406	0.869	0.790
EMD_Ave	0.844	0.764	0.852	0.407	0.886	0.812
EMD_Min	0.813	0.741	0.845	0.406	0.868	0.787

**Table 3:** Performance comparisons of different evaluation criteria on the SHREC 2013 dataset. The best performance indicators are marked as bold.

Methods	NN	FT	ST	E	DCG	AP
CDMR [13]	0.279	0.203	0.296	0.166	0.458	0.250
SBR-VC [5]	0.164	0.097	0.149	0.085	0.348	0.116
SP [32]	0.017	0.016	0.031	0.018	0.240	0.026
FDC [5]	0.053	0.038	0.068	0.041	0.279	0.051
Siamese [20]	0.405	0.403	0.548	0.287	0.607	NA
DCML [17]	0.650	0.634	0.719	0.348	0.766	NA
LWBR [21]	0.712	0.725	0.785	0.369	0.814	NA
SEM [19]	0.823	**0.828**	**0.860**	0.403	0.884	NA
OURS	**0.849**	0.772	0.858	**0.410**	**0.888**	**0.817**

**Table 4:** Performance comparisons of different evaluation criteria on the SHREC 2014 dataset. The best performance indicators are marked as bold.

Methods	NN	FT	ST	E	DCG	AP
BF-fGALIF [8]	0.115	0.051	0.078	0.036	0.321	0.044
CDMR [13]	0.109	0.057	0.089	0.041	0.328	0.054
SBR-VC [5]	0.095	0.050	0.081	0.037	0.319	0.050
BOF-JESC (Words800-VQ) [33]	0.086	0.043	0.068	0.030	0.310	0.041
Siamese [20]	0.239	0.212	0.316	0.140	0.496	NA
DCML [17]	0.272	0.275	0.345	0.171	0.498	NA
LWBR [21]	0.403	0.378	0.455	0.236	0.581	NA
MVPR [34]	0.546	0.506	0.642	0.301	0.715	0.543
SEM [19]	0.804	**0.749**	**0.813**	**0.395**	0.870	NA
OURS	**0.830**	0.708	0.807	0.384	**0.871**	**0.745**
